# Reliability, agreement and variability of a markerless computer vision algorithm for equine gait analysis under field conditions

**DOI:** 10.1111/evj.70109

**Published:** 2025-11-04

**Authors:** Karsten Key, Katja Berg, Jakob Kirkegaard, Kristian Ringkjær Andresen, Sabrina Skov Hansen

**Affiliations:** ^1^ Keydiagnostics Fredensborg Denmark; ^2^ iKeyVet Fredensborg Denmark; ^3^ Department of Veterinary Clinical Sciences University of Copenhagen Copenhagen Denmark

**Keywords:** equine lameness, gait analysis, groundline, horse, Maxdiff, Mindiff, vision‐based algorithm

## Abstract

**Background:**

Computer vision‐based algorithms offer accessible alternatives for equine gait analysis but require thorough assessment under diverse conditions.

**Objectives:**

To evaluate a proprietary vision‐based algorithm's reliability in measuring vertical displacement signals (VDS) at the eye, withers and croup, alongside groundline estimation, for horses trotting on straight lines and circles under field conditions.

**Study Design:**

Cross‐sectional comparative study evaluating agreement, variability and reliability of a markerless computer vision algorithm.

**Methods:**

We obtained 67 handheld iPhone recordings from 37 horses. A vision‐based algorithm and independent manual annotation produced 2D anatomical keypoints on all frames of the recordings, which were processed to estimate a groundline and compute VDS and stride‐based maxima (Maxdiff) and minima (Mindiff) vertical differences. Mean signed error (MSE), mean absolute error (MAE) and Bland–Altman plots were used to compare detected and annotated data.

**Results:**

The frame level vertical keypoint accuracy was 4.5 mm (eye), 5.5 mm (croup) and 11.8 mm (withers), and the manual annotation error was averaged at 2.7 mm. At the stride level (*n* = 1556), the overall mean absolute errors (MAEs) for both Maxdiff and Mindiff were 4.3 mm. The eye keypoint exhibited the lowest errors (2.9 mm Maxdiff, 3.0 mm Mindiff), while the withers error was 5.5 mm for both Maxdiff and Mindiff, and the croup showed 4.3 mm (Maxdiff) and 4.4 mm (Mindiff). Trial‐level (*n* = 67) analysis, with below optimal number of strides per trial in this study, revealed lower overall absolute differences (Eye: 2.3 mm, Withers: 3.7 mm, Croup: 2.7 mm), indicating consistent performance across multiple strides. Subjective lameness scoring aligned with objective measures with some variation.

**Main Limitations:**

Groundline estimation accuracy was stress‐tested on treadmill data in another study. Further clinical comparison with established gait analysis systems is recommended.

**Conclusions:**

The algorithm robustly measured vertical displacements under varied conditions.

## 1 INTRODUCTION

Accurate gait analysis is important for diagnosing and managing equine lameness effectively. Traditional methods, such as subjective visual assessments by trained clinicians, are extremely valuable but can be biased and are prone to intra‐ and inter‐observer variability.^1^, ^2^, ^3^, ^4^, ^5^ Although advanced technologies, such as force plates, inertial measurement units and optical motion capture systems exist,^6^, ^7^, ^8^, ^9^ they often require specialised, expensive equipment and controlled conditions. These constraints limit their practicality in clinical field practice.

**FIGURE 1:**
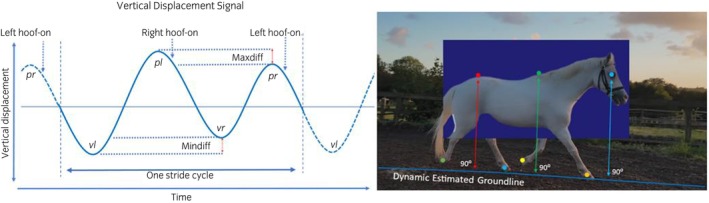
Left: example of the vertical displacement (cyclical) signal (VDS) from eye, withers or croup. A stride cycle is defined as: the minimum displacement after left hoof‐on (valley left=vl) → maximum displacement after left hoof‐off (peak left=pl) → minimum displacement after right hoof‐on (valley right=vr) → maximum displacement after right hoof‐off (peak right=pr). Metrics for lameness quantification are calculated as the difference between the two minima (Mindiff) and the two maxima (Maxdiff) per stride. This example has a Mindiff and a Maxdiff deficit on the right leg. Right: the vertical displacements (red, green, blue arrows) are measured in the 2D sagittal plane of the horse (blue square), when seen from the side, as the distance in pixels from the dynamically estimated groundline (blue) to the keypoints. The pixel difference is calibrated to a metric value, knowing the metric withers height.

In recent years, objective gait analysis and biomechanics have gained significant attention.^10^ It is widely acknowledged that tracking the cyclical vertical displacements (Figure 1) of key anatomical locations in trot, particularly the head, withers and pelvis, is useful in measuring overall gait symmetry and detecting lameness.^6^, ^7^, ^11^, ^12^, ^13^, ^14^, ^15^, ^16^, ^17^ The indicators of impact and push‐off asymmetry identified within each stride cycle are especially important and are quantified by the difference in minimum displacement (Mindiff) and the difference in maximum displacement (Maxdiff) from the left and right halves of the stride cycle. These and other metrics are employed by various systems, including inertial measurement units, optical motion capture setups and computer vision techniques, underscoring their essential role in data‐driven gait assessment.^6^, ^7^, ^11^, ^12^, ^14^, ^15^, ^16^, ^17^, ^18^ Recent advancements in computer vision and deep learning have led to the development of markerless 2D motion capture systems.^19^, ^20^, ^21^, ^22^, ^23^, ^24^, ^25^ By analysing video data and identifying key anatomical landmarks, such as the head, withers and croup, as pixel coordinates in each frame, these systems can extract crucial movement parameters, including the two‐dimensional cyclical Vertical Displacement Signal (VDS), thereby offering a more accessible alternative for both veterinarians and horse owners 25. However, their reliability under diverse field conditions remains insufficiently understood.

The many different systems for objective gait analysis either employ methods that derive a VDS measured from the ground (ground truth method) as in this study or more commonly analyse the relationship between the first and second harmonics of the VDS.^6^, ^7^, ^11^, ^12^, ^14^, ^15^, ^16^, ^17^, ^18^, ^25^ This study aimed to evaluate the agreement, variability and reliability of a custom, markerless computer vision algorithm against manual annotation for equine gait analysis in horses trotting on a straight line and on a circle under field conditions. Comparing algorithm‐detected keypoints against a manually annotated control dataset is considered the industry standard for validating the accuracy and precision of 2D computer vision deep neural network algorithms.^20^

## 2 MATERIALS AND METHODS

We conducted a comparative study using manually annotated keypoints as a reference. In this approach, an experienced annotator manually labelled the eye, withers, croup and hooves in each video frame, creating a reference dataset against which to assess the algorithm's precision in annotating the correct keypoints used for extracting the VDS.

The two datasets of annotated keypoints, one created by manually placing the keypoints (reference set) and one generated by the computer vision algorithm (test set), were downstream processed identically. The algorithm estimated a dynamic groundline frame‐by‐frame, using the hoof keypoints. The vertical displacement of the eye, withers and croup was measured in pixel distance relative to this, creating a raw VDS (Figure 1). The VDS signal was calibrated to millimetres, knowing the withers height of the horses and filtered for low‐frequency noise.^17^ Following Keegan et al.,^12^ we computed the maximum (Maxdiff) and minimum (Mindiff) differences in VDS, measured in millimetres, within each segmented stride for the eye, withers and croup (Figure 1). By comparing these outcome measures between the two annotation methods, we directly assess the algorithm's agreement in VDS signal extraction, focusing on the Mindiff and Maxdiff metrics. Finally, we compare the algorithm's performance under both straight‐line and lunging conditions. This study is part of two studies testing the algorithm, the other being a groundline estimation study, where horses trotting on a treadmill were filmed from various angles to stress‐test the accuracy and precision of the estimated groundline compared with the real groundline [submitted for peer review].

### 2.1 Study population, equipment and data collection

**FIGURE 2:**
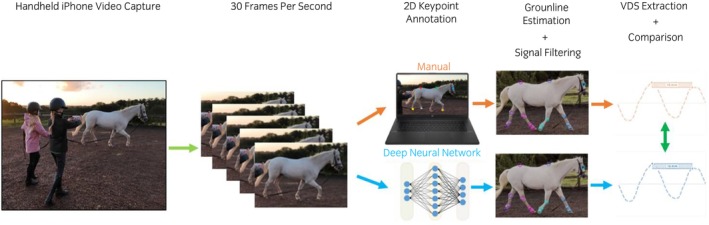
Experimental setup. The study population (*n* = 37 horses and n = 67 recordings) was recorded from the side while lungeing (from the centre) or on a straight line with a handheld iPhone recording at HD, 30 FPS. The frames were keypoint annotated manually and independently by a trained deep neural network. The resulting 2D keypoints led to groundline estimation and VDS filtering, in the exact same method. The resulting VDS signals were stride split, and the matching strides were compared statistically by comparing Mindiff and Maxdiff. The VDS curves on the right illustrate matching strides and a Maxdiff deficit of the right leg as an example.

Sixty‐seven recordings were collected from a sample of 37 horses of varying breeds (Warmblood, Thoroughbred, pony, Icelandic and coldblood), coat colours (grey, bay, black, red, pinto) and withers heights (130–175 cm) at both indoor and outdoor locations. Recordings were handheld in landscape mode with different iPhone models (Model 13 Pro and 15 Pro, iOS‐Version 15.5.1, Apple Inc). The two iPhone models were used at random, without choosing a specific model for each recording. The setup is illustrated in Figure 2. The study population was a convenience sample of horses undergoing clinical examination by one author (K.K.). None of the horses contributed data to the deep neural network training. Forty‐six recordings of various lengths were obtained while the horses were lunged in either direction, and 21 recordings were obtained from straight‐line trotting. The surface underfoot was gravel, sand or fibre‐sand without special levelling. Each recording of random length included between 2 and 17 detected segmented strides. As the process of manual annotation is labour‐intensive, emphasis was placed on capturing a diverse range of horses and recording conditions, rather than extending the recording length, leading the comparative analysis to focus on stride‐based differences rather than trial‐based comparisons. All horses were examined in a clinical setting without any modification to their routine handling, and recordings were made using the commercially available app as part of their standard clinical examination. All 67 recordings were independently and blindly evaluated by an experienced equine lameness specialist, certified by both the International Society of Equine Locomotor Pathology (ISELP) and the Equine Gait Analysis Society (EGAS). Subjective lameness was graded using a subjective 0–5 scale, where 0 is sound and 5 is non‐weightbearing, with the use of half grades (e.g., 1.5) permitted.

As the data collection was non‐invasive and part of routine clinical care, no national ethical approval was required. Written owner consent was obtained prior to inclusion in the study.

### 2.2 Manual annotation

Manual annotations were performed for comparison with the algorithm‐detected keypoints, as manual annotation is considered a gold standard reference for computer vision and human/animal deep neural pose estimation.^20^, ^21^ The keypoints annotated manually for each frame of the recordings were eye, withers, croup and hooves. The fetlock and carpus/hock keypoints detected by the algorithm were not used for calculating the VDS and were consequently not detected manually. A total of seven keypoints on each of the 14,238 frames resulted in 99,666 manual keypoint annotations. All keypoints were annotated by the same person (K.B.) using a custom annotation tool (RealHorse®). This provided a reference dataset for assessing the agreement of the algorithm's detected keypoints and the resulting VDS.

### 2.3 Detected keypoints

Core analysis for algorithm‐detected keypoints was based on a trained deep neural network capable of identifying keypoints on the horse from a side view (RealHorse®). This model was trained on a large dataset (231 videos, 65,000 frames and more than 1 mill. manually annotated keypoints) including a diverse cohort of horse types trotting on a straight line, while being lunged and one horse on a treadmill. None of the horses in the training set were used in this study. The primary keypoints were the eye, withers, back and croup, along with three keypoints on each leg: hoof, fetlock and carpus/hock.

The network was applied to every frame in each recorded video. If a keypoint was not detected in a particular frame, the stride detection process including that frame failed, resulting in minor variations in the detected strides among different keypoints. In one horse, severe lameness in a front leg led to missing double minima and maxima in the VDS within the only two recorded strides, causing a missing trial for the eye keypoint. All detected strides and trials were included in the study.

The algorithm detects keypoint positions with subpixel precision, whereas manual annotation was limited to integer pixel values. This introduces a quantisation effect, where even correct identification of the same anatomical feature may result in small discrepancies due to rounding. The maximum quantisation error is approximately (√2)/2 equalling 0.71 pixels, which translates to 1.4–2.1 mm in physical space based on the typical pixel resolution (2–3 mm per pixel depending on horse size and distance to camera).

### 2.4 Signal filtering and symmetry metrics computation

For both the algorithm‐detected and manually annotated keypoints, the same downstream processes were carried out with either of the two keypoint datasets (Figure 2). The groundline was dynamically estimated frame‐by‐frame from the hoof keypoints by identifying ground contact (stance phase) and off‐the‐ground (swing phase) intervals.^6^, ^7^, ^8^, ^11^, ^26^ The recordings were stabilised based on the groundline, and the frames rectified by the algorithm before further analysis.

The recordings were then calibrated according to the horse's known metric withers height, allowing vertical motion to be expressed in millimetres. In the rectified recordings, the relevant keypoints (eye, withers and croup) were evaluated with respect to vertical movement relative to the groundline to generate the VDS (Figure 1). The raw signal combined frequencies from pure trotting motion with noise stemming from camera movement, occasional horse erratic motion and keypoint fluctuation. To isolate the relevant VDS frequency components, the expected trotting frequency was robustly estimated based on stride frequency. A 3rd order Butterworth high‐pass filter was applied with a cut‐off set just below each horse's algorithm estimated trot frequency, thereby preserving the core trotting signal while removing low‐frequency noise.^17^, ^27^

Once the filtered VDS was obtained, individual strides were split based on expected peaks and valleys, guided by hoof keypoints for stance and swing timing^16^ as well as the manual or algorithms identification of left versus right. To quantify potential asymmetry, two metrics—Maxdiff and Mindiff—were calculated for each stride in both the algorithm‐detected and manually annotated datasets for eye, withers and croup. Maxdiff was defined as the difference between consecutive maxima in the VDS, while Mindiff was the difference between consecutive minima^12^ (Figure 1). These metrics formed the basis for evaluating the algorithm's precision and agreement with the manual annotations.

### 2.5 Data analysis

#### 2.5.1 Frame‐level keypoint analysis

To assess the vertical accuracy between algorithm‐detected and manually annotated keypoints at the frame level, a dedicated frame‐by‐frame comparison was performed. This analysis focused on the eye, withers and croup keypoints across all annotated video frames (*n* = 14,238), which are used in the computation of the Vertical Displacement Signal (VDS). The vertical accuracy of the hoof keypoints and the consequently estimated groundline, also used when generating the VDS, is currently being assessed in our group. Keypoint comparisons were made prior to any signal filtering.

Each frame included one set of manually annotated keypoints and a corresponding set of algorithm‐detected keypoints, both expressed in pixel coordinates. For each keypoint and frame, the vertical distance between the keypoint pairs was calculated and converted from pixel coordinates to millimetres, knowing the withers' height. Signed differences (algorithm minus manual) and absolute differences were then computed to assess agreement.

Histograms were generated to visualise the distribution of signed and absolute errors for each keypoint. Additionally, the mean bias, 95% limits of agreement, mean absolute error (MAE) and 95% confidence intervals were calculated. The analysis also examined the influence of recording conditions (straight‐line vs. circle) and anatomical variation (presence or absence of visibly moving mane hair over the withers) on agreement, along with additional variables such as surface, coat colour, breed, recording environment (outdoor vs. indoor) and iPhone model.

#### 2.5.2 Manual annotation variability

To assess the variability of manual keypoint annotation, we evaluated the vertical agreement between algorithmically detected keypoints and two independent rounds of manual annotation. A representative subset of 25 recordings, 10 straight‐line and 15 circle, was selected from the main dataset of 67 recordings. For each, a single stride was identified near the midpoint of the video. Existing algorithmic and manual annotations were supplemented by a second manual reannotation of the selected strides, performed independently by the same annotator without access to the original annotation.

Vertical distances from each keypoint to the groundline were measured in pixels and converted to millimetres using the known withers height of each horse. For keypoint‐level comparisons in this substudy, a single groundline, estimated from algorithmically detected hoof keypoints, was applied consistently across all stride frames. These comparisons were made prior to any signal filtering, resulting in 2883 data points assessing the vertical position of the eye, withers and croup across annotation methods.

For stride‐level analysis, the groundline was independently estimated for each annotation method, and vertical displacement signals (VDS) for the eye, withers and croup were extracted accordingly. MaxDiff and MinDiff values were obtained from the filtered VDS for each stride, yielding a pooled dataset of 146 comparisons. This allowed the evaluation of annotation consistency at the stride level.

#### 2.5.3 Groundline estimation comparative analysis

The groundline estimated with the algorithm derived from hoof keypoints was compared with the groundline estimated with manually annotated hoof keypoints, and videos were generated with the two groundlines for visual inspection. Angle differences were calculated for each frame (*n* = 14,238); mean signed angle error and mean absolute angle errors, as well as distribution histograms, were plotted.

#### 2.5.4 Stride‐ and trial‐based comparative analysis

Descriptive statistics, with a focus on the difference of agreement between Maxdiff and Mindiff from manually annotated versus algorithm‐detected key points, including mean absolute error (MAE), mean signed error (MSE) and sample standard deviation (SD), were computed to summarise the algorithm's performance.

Bland–Altman^28^, ^29^ and scatterplots were generated to evaluate the agreement between manually annotated and algorithm‐detected data, focusing on stride‐based and secondary trial‐based comparisons. Absolute and signed error distributions were analysed using histograms to identify clustering and variability trends. These analyses were conducted separately for eye, withers and croup keypoints, as well as for lungeing and straight‐line conditions.

#### 2.5.5 Relationship between trial error and number of strides

When a stride‐level test exhibits normally distributed errors around zero, the relationship between the number of strides (*n*) and the trial‐based mean absolute error (MAE) for Maxdiff and Mindiff follows the principle of error reduction through averaging.^30^ Specifically, random errors tend to cancel each other out when averaged over multiple measurements, reducing the overall trial MAE.

In practice, the mean of individual stride errors (i.e., at stride level) is calculated for each trial, and the trial‐level MAE is then derived from these averages. The reduction in error occurs because the standard deviation of the error distribution (*σ*) is scaled down by the square root of the number of strides (*n*) used in the calculation. This statistical property ensures that as more strides are recorded within a trial, the overall error decreases, in this study improving agreement between annotated and detected data. A systematic bias (*b*) introduces a non‐random component, shifting the error distribution away from zero. To account for both random errors and systematic bias, the theoretical trial‐based MAE can be computed as:

$$\text{Trial}\;\mathrm{MAE}\left(n\right)=\surd \left({\left(\left(\sigma \times \surd \left(2/\pi \right)\right)/\surd n\right)}^{2}+b^{2}\;\right),$$

where *σ*: the standard deviation of stride‐level errors (random error component). *n*: the number of strides per trial. *b*: the bias (mean of signed error).

This formula accounts for both the random error reduction through averaging and the impact of systematic bias.

## 3 RESULTS

### 3.1 Subjective lameness evaluation

Subjective lameness scoring of the 67 recordings of trotting horses yielded grades ranging from 0 to 2.5 for the forelimbs and from 0 to 1.5 for the hindlimbs.

### 3.2 Frame‐level keypoint agreement

**FIGURE 3:**
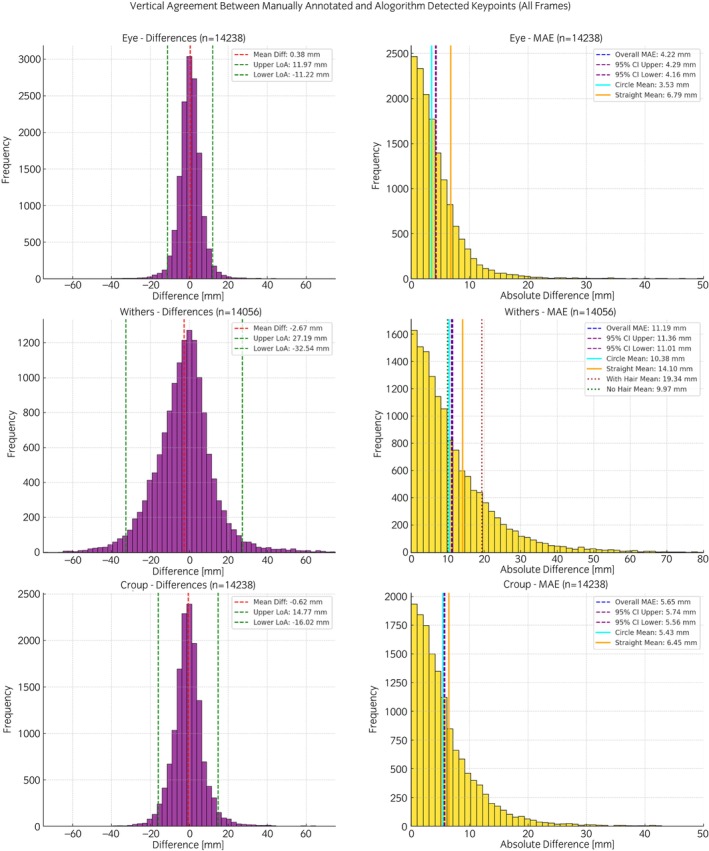
Histograms illustrating the vertical agreement between manually annotated and algorithm‐detected keypoints (eye, withers and croup) based on all video frames (*n* = 14,238). The histograms of signed differences (left panels) show mean bias (solid red line) with 95% limits of agreement (green dashed lines), demonstrating minimal systematic bias. Histograms of mean absolute errors (MAE; right panels) show overall MAE (blue dashed lines) with 95% confidence intervals (purple dashed lines). Vertical coloured lines indicate condition‐specific means for recordings on circles (cyan solid line), straight lines (orange solid line), and for withers, with hair (brown dotted line) or without hair (dark green dotted line), illustrating the effect of these conditions on keypoint detection precision.

Frame‐level vertical agreement between algorithm‐detected and manually annotated keypoints was evaluated for the eye, withers and croup across all 14,238 frames. Histograms of signed differences showed minimal systematic bias for the eye and croup keypoints. The withers keypoint had a mean signed difference of −2.7 mm compared with manual annotation and wider 95% limits of agreement than the other keypoints (Figure 3).

Mean absolute error (MAE) was lowest for the eye (4.54 mm, 95% CI: 3.97–5.11 mm), followed by the croup (5.57 mm, 95% CI: 4.96–6.18 mm) and highest for the withers (11.84 mm, 95% CI: 9.81–13.87 mm) (Figure 3, right panels). Recordings with visibly moving mane hair over the withers had a higher withers MAE (19.96 mm) than those without (9.89 mm).

MAEs were also higher in straight‐line recordings compared with recordings on a circle across all keypoints. For example, the eye keypoint had a mean MAE of 6.75 mm on straight lines compared with 3.53 mm on the circle. No significant effect of surface, coat colour, breed, recording environment (indoor vs. outdoor) or iPhone model was observed on the level of agreement.

### 3.3 Manual annotation variability

#### 3.3.1 Keypoint‐level manual annotation variability

**FIGURE 4:**
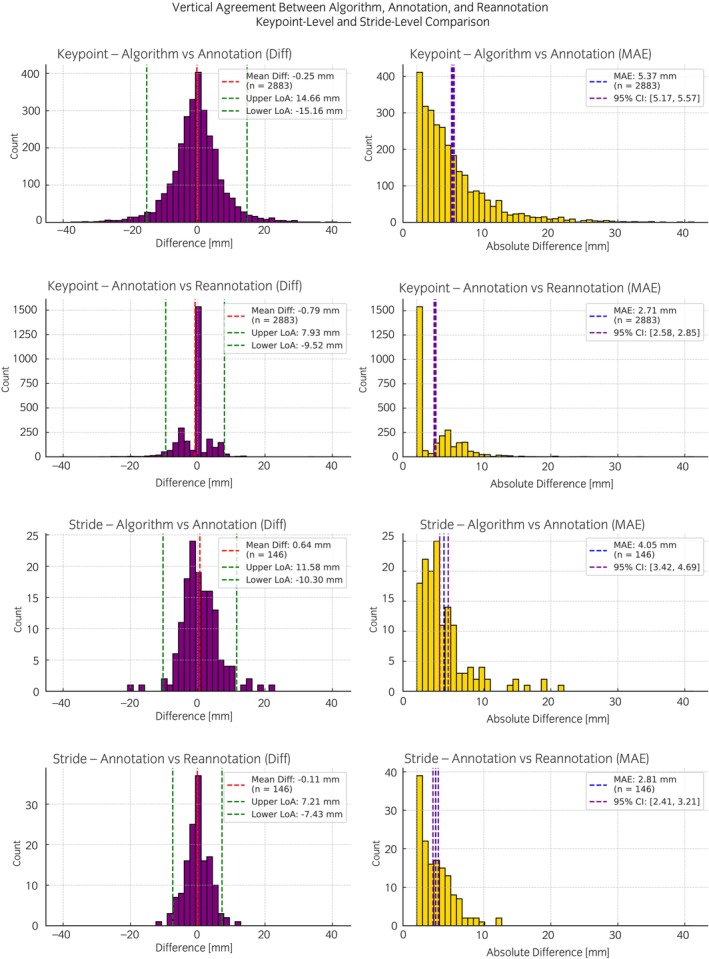
Top panels: vertical agreement between manually annotated and algorithmically detected keypoints (*n* = 2883 frames). Left: histogram of vertical position differences (mm) with mean difference and 95% limits of agreement (LoA). Right: histogram of mean absolute errors (MAE) with 95% confidence intervals. Comparisons are shown for both algorithm vs. annotation and annotation vs. reannotation. Bottom panels: vertical agreement at stride‐level Maxdiff and Mindiff frames for eye, withers and croup across 25 horses (*n* = 146). Left: differences in vertical keypoint position relative to the groundline. Right: MAE distributions with 95% confidence intervals.

Figure 4 (top panels) displays the distribution of vertical position differences for the eye, withers and croup keypoints across all stride frames in the subset (*n* = 2883). The mean difference between algorithm and manual annotation was −0.25 mm, while the mean difference between annotation and reannotation was −0.71 mm. The overall mean absolute error (MAE) was 5.37 mm (95% CI: [5.17, 5.57]) for algorithm vs. annotation, and 2.71 mm (95% CI: [2.58, 2.85]) for annotation vs. reannotation.

#### 3.3.2 Stride‐level manual annotation variability

Figure 4 shows the results for the stride‐level comparison based on 146 pooled Maxdiff and Mindiff values for the eye, withers and croup from the subset. The MAE between the algorithm and annotation was 4.05 mm (95% CI: [3.42, 4.69]), while annotation vs. reannotation yielded an MAE of 2.81 mm (95% CI: [2.41, 3.21]).

### 3.4 Groundline estimation results

The analysis of groundline angle errors was performed on a dataset of 14,238 frames to evaluate the level of agreement between groundlines estimated with either of the two keypoint annotation methods. The signed groundline angle error exhibited a mean bias of 0.10°, with 95% limits of agreement ranging from −0.75° to 0.95°. These results suggest minimal systematic bias in the measurements. The absolute groundline angle error showed a MAE of 0.27°, with a narrow 99% confidence interval of 0.26° to 0.27°.

**FIGURE 5:**
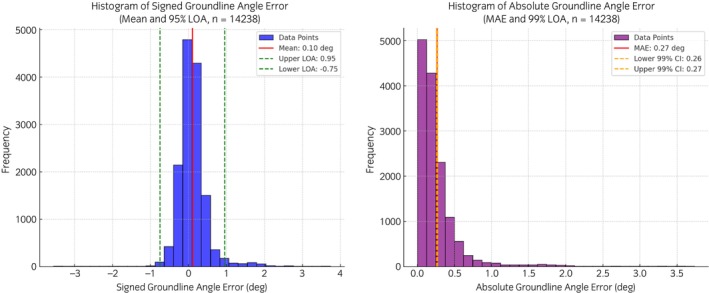
Histograms depicting the signed and absolute groundline angle errors for *n* = 14,238 observations. Left: histogram of signed groundline angle error, showing a mean error of 0.10° (red line) and 95% limits of agreement (green dashed lines) ranging from −0.75° to 0.95°. Right: histogram of absolute groundline angle error, with a mean absolute error (MAE) of 0.27° (red line) and a 99% confidence interval (orange dashed lines) between 0.26° and 0.27°. The distribution highlights high agreement in the groundline angle measurements.

Most errors were close to zero, indicating high agreement in groundline estimation between algorithm‐detected hoof keypoints and those based on manual annotation (Figure 5).

### 3.5 Stride detection and missing data

Of the 67 recordings, the number of detected strides was as follows: Eye (*n* = 514), withers (*n* = 518) and croup (*n* = 524). Based on the average frames per stride, we estimated a maximum of approximately 550 detectable strides, accounting for the typical average loss of one stride per recording due to incomplete cut stride cycles. Consequently, under field conditions, the stride detection failure rates were estimated to be about 6% for all keypoints. Visual inspection of the videos suggests that most detection failures occurred because the algorithm required a few frames to locate all keypoints initially. If the algorithm took more than, on average, 0.7 s (approximately the duration of one stride) to identify these keypoints, the recording would lose more than the typical one stride.

One horse was severely lame in a front leg and did not exhibit the characteristic double minima and maxima in the vertical displacement signal (VDS) for the eye keypoint. As a result, the eye keypoint was not successfully stride detected for that horse, leading to a missing trial. However, because this very lame horse was trotted for only very few strides, it did not affect the eye keypoint's overall stride detection failure rate.

### 3.6 Stride‐based results

Stride‐based results are presented in Tables 1 and 2. The algorithm achieved an overall stride‐based MAE of 4.26 mm for Maxdiff and 4.29 mm for Mindiff. The lowest MAE values were observed for the eye, with 2.93 mm (Maxdiff) and 3.03 mm (Mindiff) with less variability for circle versus straight‐line. The withers exhibited slightly higher MAE values of 5.52 mm (Maxdiff) and 5.46 mm (Mindiff), with no significant variability on straight‐line versus circle. The croup demonstrated similarly low MAE values of 4.33 mm (Maxdiff) and 4.38 mm (Mindiff) with similar variability across circle and straight‐line.

**TABLE 1:** Stride‐based analysis for Maxdiff. number of strides (n), mean absolute error (MAE, including 95% confidence intervals), mean signed error (MSE) and standard deviation (SD).

Keypoint	*n* (strides)	MAE Maxdiff (mm)	MAE 95% CI	MSE	SD
All keypoints	1556	4.26	[3.97; 4.55]	0.11	5.85
Eye	514	2.93	[2.57; 3.29]	0.02	4.17
Eye straight	92	4.77	[3.44; 6.10]	−0.12	6.50
Eye circle	422	2.53	[2.19; 2.86]	0.05	3.47
Withers	518	5.52	[4.91; 6.14]	0.58	7.13
Withers straight	92	5.08	[3.80; 6.35]	0.62	6.24
Withers circle	426	5.62	[4.93; 6.32]	0.57	7.32
Croup	524	4.33	[3.83; 4.83]	−0.28	5.83
Croup straight	94	4.59	[3.40; 5.78]	−0.47	5.89
Croup circle	430	4.27	[3.72; 4.82]	−0.23	5.83

**TABLE 2:** Stride‐based analysis for Mindiff. number of strides (*n*), mean absolute error (MAE, including 95% confidence intervals), mean signed error (MSE) and standard deviation (SD).

Keypoint	*n* (strides)	MAE Mindiff (mm)	MAE 95% CI	MSE	SD
All keypoints	1556	4.29	[4.00; 4.58]	0.17	5.81
Eye	514	3.03	[2.69; 3.37]	0.09	3.93
Eye straight	92	4.75	[3.57; 5.92]	0.99	5.76
Eye circle	422	2.65	[2.33; 2.97]	−0.10	3.38
Withers	518	5.46	[4.85; 6.06]	0.43	7.03
Withers straight	92	6.19	[4.63; 7.75]	0.49	7.62
Withers circle	426	5.30	[4.65; 5.95]	0.63	6.89
Croup	524	4.38	[3.86; 4.89]	−0.00	6.02
Croup straight	94	5.06	[3.69; 6.42]	0.63	6.74
Croup circle	430	4.23	[3.68; 4.78]	0.13	5.85

Bland–Altman plots in stride‐based evaluations showed narrow limits of agreement, indicating minimal bias between the manually annotated and algorithm‐detected values across all keypoints. Supplementary material includes Eye, Withers and Croup Mindiff and Maxdiff stride error data comparing algorithm‐detected and manually annotated values (Figures [Link], [Link]).

### 3.7 Trial‐based results

Trial‐based analysis aggregated Maxdiff and Mindiff values across individual recordings (*n* = 67), focusing on differences of absolute mean values within each trial (MAE). Table 3 summarises the trial‐based results for eye, withers and croup keypoints, overall and across lungeing and straight‐line conditions.

**TABLE 3:** Trial‐based analysis of error for Maxdiff and Mindiff combined.

Keypoint	Recording	*n* (trials)	Strides/trial (mean)	MAE (mm)	MAE 95% CI	SD	MSE
Eye	Overall	66	7.79	2.25	[1.73; 2.78]	3.07	0.42
Eye	Circle	45	9.38	1.68	[1.21; 2.14]	2.25	0.09
Eye	Straight	21	4.38	3.49	[2.19; 4.79]	4.29	1.12
Withers	Overall	67	7.73	3.65	[2.89; 4.41]	4.51	0.63
Withers	Circle	46	9.26	3.50	[2.61; 4.40]	4.38	0.88
Withers	Straight	21	4.38	3.97	[2.52; 5.42]	4.8	0.06
Croup	Overall	67	7.82	2.66	[2.01; 3.30]	3.81	−0.49
Croup	Circle	46	9.35	2.56	[1.75; 3.36]	3.94	−0.31
Croup	Straight	21	4.48	2.88	[1.82; 3.94]	3.51	−0.90

The eye demonstrated the lowest absolute difference for Maxdiff and Mindiff under circular trajectories, with mean absolute differences of 1.68 mm and an overall difference of 2.25 mm.

Slightly higher differences were observed for both withers Maxdiff and Mindiff, where circular trajectories showed slightly lower variability compared with straight‐line conditions. Withers' overall absolute differences were 3.65 mm. The croup showed similar low absolute differences across all trajectories (overall 2.66 mm) and consistent results in circular and straight‐line conditions.

**FIGURE 6:**
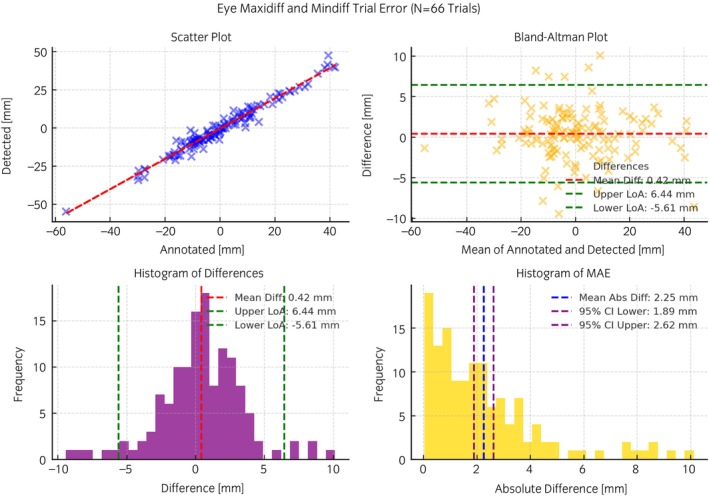
Eye trial mean Max‐/Mindiff data for 66 trials comparing algorithm‐detected and manually annotated values. The scatter plot (top‐left) shows strong alignment along the line of equality, while the Bland–Altman plot (top‐right) indicates 95% limits of agreement at −5.61 to +6.44 mm. The histogram of signed differences (bottom‐left) reflects a bias of 0.42 mm, and the histogram of absolute differences (bottom‐right) shows a mean absolute error of 2.25 mm, collectively illustrating a generally strong agreement.

**FIGURE 7:**
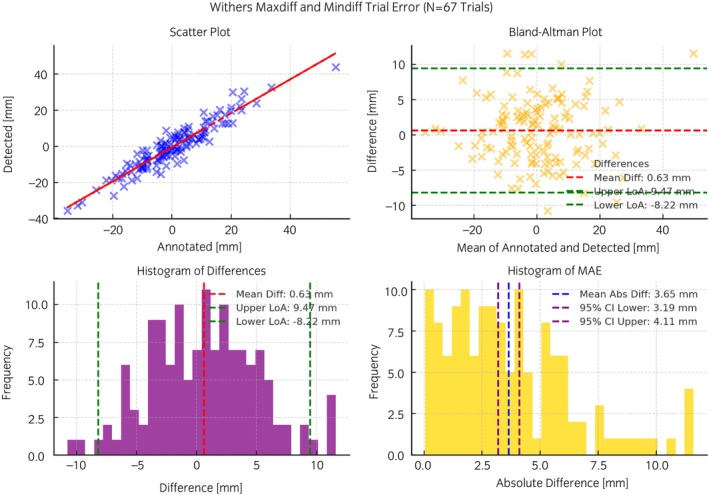
Withers trial mean Max‐/Mindiff data for 67 trials comparing algorithm‐detected and manually annotated values. The scatter plot (top‐left) shows good alignment along the line of equality, while the Bland–Altman plot (top‐right) indicates 95% limits of agreement at −8.22 to +9.47 mm. The histogram of signed differences (bottom‐left) reflects a bias of 0.63 mm, and the histogram of absolute differences (bottom‐right) shows a mean absolute error of 3.65 mm, highlighting good agreement.

**FIGURE 8:**
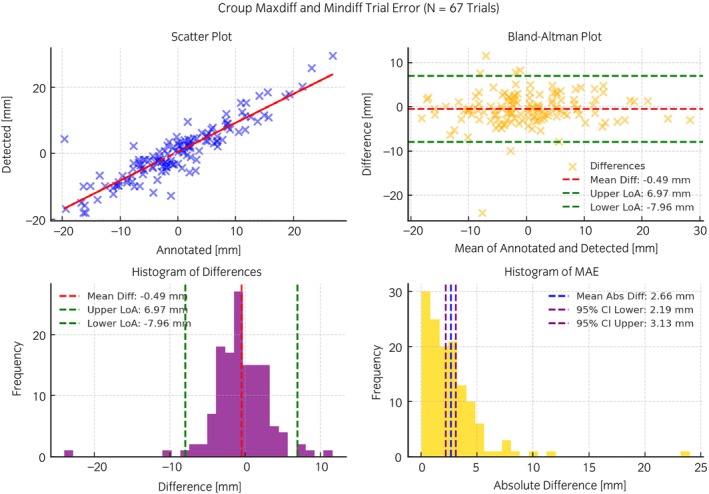
Croup trial mean Max‐/Mindiff data for 67 trials comparing algorithm‐detected and manually annotated values. The scatter plot (top‐left) shows good alignment along the line of equality, while the Bland–Altman plot (top‐right) indicates 95% limits of agreement at −7.96 to +6.97 mm. The histogram of signed differences (bottom‐left) reflects a bias of −0.49 mm, and the histogram of absolute differences (bottom‐right) shows a mean absolute error of 2.66 mm, demonstrating strong agreement.

Bland–Altman plots in trial‐based evaluations showed narrow limits of agreement (Figures 6, 7, 8), indicating minimal bias and good agreement between the manually annotated and algorithm‐detected values across all keypoints.

#### 3.7.1 Relationship between trial error and number of strides

For this study, the stride‐level aggregated Maxdiff and Mindiff standard deviations (*σ*) for overall eye, withers and croup were 4.05, 7.08 and 5.93 mm, respectively. The corresponding biases (*b*) were 0.06 mm for eye, 0.51 mm for withers and −0.14 mm for croup.

**FIGURE 9:**
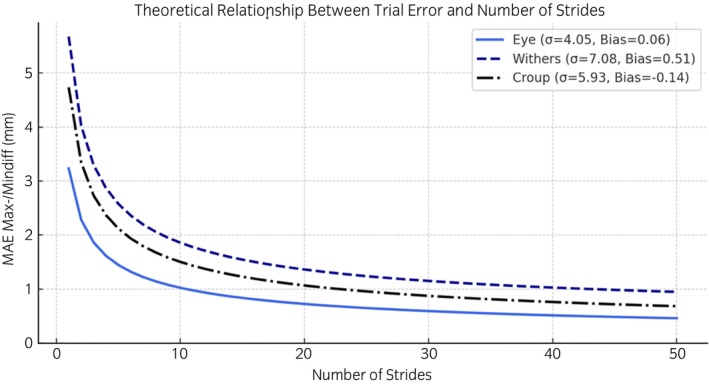
Theoretical relationship between trial error and number of strides, based on stride error data from this study. The plot illustrates the theoretical trial error, based on the stride‐level mean absolute error (MAE) for Max‐ and Mindiff for the keypoints: eye, withers and croup. The MAE is calculated assuming that the errors follow a normal distribution. The relationship between the standard deviation (*σ*) of the stride‐level errors and the expected MAE is given by the formula: Trial MAE(*n*) = √ (((*σ* × √(2/*π*))/√*n*)^2^ + *b* ^2^). Using this formula, the starting Stride MAE for each keypoint is calculated based on the observed standard deviation of stride‐level errors and the bias. The theoretical trial error then decreases with the number of strides. Key starting Stride MAE values: eye: Stride MAE = 3.23 mm, withers: Stride MAE = 5.65 mm, croup: Stride MAE = 4.73 mm.

Using these values, Figure 9 illustrates the bias‐adjusted theoretical relationship between the number of strides (*n*) and the predicted trial‐based MAE. To compare with an actual trial MAE value, the actual Eye trial MAE was observed to be 2.25 mm, with an average of 7.79 strides per trial, slightly higher than the theoretical curve would suggest. However, trials with fewer total strides contributed disproportionately to the overall mean absolute error, causing a larger deviation from the expected theoretical trend. This study was primarily designed to test for stride‐based error and, as such, the mean number of strides per trial (Table 3) is lower than optimal, as seen in Figure 9.

## 4 DISCUSSION

This study compared algorithm‐detected and manually annotated keypoints for measuring equine gait asymmetry under field conditions. In addition to stride‐level symmetry metrics, we assessed the frame‐level agreement between algorithm‐detected and manually annotated keypoints. We also evaluated the algorithm's ability to estimate the groundline using standard handheld video. Manual annotation served as the reference standard, following common practice in computer vision and human/animal 2D pose‐estimation research,^20^, ^21^ although its use as ground truth in equine gait analysis is novel and requires further validation. To support this approach, manual reanotation variability has been quantified.^31^

The findings demonstrate that the deep neural network can reliably identify the eye, withers, croup and hoof landmarks in horses trotting on straight lines or circles. It generates vertical displacement signals (VDS) and asymmetry metrics (Maxdiff and Mindiff) with low mean absolute errors (MAE) compared with manually annotated data, confirming its high level of agreement with manual annotation for automated gait analysis in equine locomotion studies.

The frame‐level vertical keypoint precision analysis showed high consistency for the eye and croup keypoints, whereas the withers exhibited a small downward bias and greater variability. The withers' variability was influenced by the presence of visibly moving mane hair over the withers, and recordings on a circle yielded better overall keypoint agreement than those on a straight line. No other investigated variables (surface, breed, coat colour, recording environment or iPhone model) were found to significantly influence keypoint agreement.

The results of the manual annotation variability substudy indicate good vertical agreement between repeated manual annotations. Given the resolution of the custom‐made annotation tool, where one pixel corresponds to approximately 2–3 mm in metric units, many annotations landed on the same pixel row, resulting in a high frequency of zero difference.

Where manual annotation is subject to quantisation limitations due to its restriction to integer pixel values, the algorithm estimates keypoint locations with subpixel accuracy. This inherent resolution difference introduces a maximum possible error of 0.71 pixels, or approximately 1.4–2.1 mm, even when the same anatomical feature is correctly identified.

At stride level, annotation vs. reannotation errors were also lower than algorithm vs. annotation, although the difference was less pronounced than at keypoint level. This supports the robustness of the VDS filtering method in minimising the impact of frame‐to‐frame noise and random keypoint annotation fluctuations.

Manual annotation, while labour‐intensive, continues to serve as a dependable reference standard when evaluating the accuracy of computer vision‐based 2D systems, where body‐mounted sensors may introduce noise or tracking drift. Accurate groundline estimation is vital for 2D VDS analysis when applying a ground truth VDS method. The present study supports the algorithm's strong groundline estimation accuracy, as the groundline derived from algorithm‐detected hoof keypoints aligns closely with the groundline from manually annotated hooves (mean absolute angle error <0.3°). This stability is especially important when using a handheld camera, since this algorithm does not rely on a static setup during lungeing or straight‐line assessments.

Both stride‐ and trial‐level analyses consistently revealed minimal differences between the algorithm‐ and manually derived VDS metrics. The eye keypoint had the smallest overall stride‐based Max‐ and Mindiff MAE (3.0 mm). The withers (5.5 mm) and croup (4.5 mm) showed slightly higher errors, which remain clinically acceptable. Averaging errors at the trial level reduced differences further, reflecting the effect of averaging out random stride‐to‐stride variations.

Lungeing conditions, where the horse remains at a more constant distance and orientation from the camera, tended to yield smaller stride‐level errors than straight‐line recordings. Horses trotting away or towards the camera on a straight path, when recorded from the side, are subject to shifting angles and perspective, which can introduce greater variability. Even so, these discrepancies remained within limits acceptable for routine clinical use.

The theoretical relationship between trial error and the number of strides can be used to establish guidelines for the recommended number of recorded strides in the clinical use of the algorithm.

When compared with other published equine gait analysis systems, these results appear favourable. One recent markerless study evaluated head and pelvis 2D tracking against 3D optical motion capture but did not include the withers.^25^ This system also used an iPhone but recorded from the front and rear, estimating stride symmetry by calculating the ratio between the first and second harmonics of the vertical displacement signal (VDS), instead of using a ground‐relative measurement like in the current study. They used a setup where the iPhone was fixed on a tripod and the horse was trotting on a level concrete floor. They reported a series of stride exclusions and excluded trials with less than 10 matching strides, resulting in an average strides per trial of 29 for the head and 17 for the pelvis. They reported a trial‐level MAE of 2.2 mm for both the head and pelvis. In comparison, our trial‐level MAEs were 2.3 mm for the eye and 2.7 mm for the croup, despite having fewer and suboptimal strides per trial (average of 8) and without applying stride exclusions. They did not report their corresponding stride‐level MAE values but instead stride‐level standard deviations of 10.0 for the head and 6.0 for the pelvis. Our corresponding stride‐level errors were lower for the head (eye SD = 4.1) and comparable for the pelvis (croup SD = 5.9), indicating our system had overall lower head‐related stride‐based vertical displacement errors and similar pelvis errors.

Most comparative validation studies for objective gait analysis systems primarily report trial‐level agreement with other objective measurement systems.^25^, ^32^, ^33^ Such studies should be interpreted with caution because, with enough strides, random stride‐to‐stride errors tend to cancel each other out,^30^ which can create a misleading impression of precision if one only examines aggregated trial metrics. Stride‐level reporting, preferably without stride exclusion, is therefore crucial for providing a clear and comparable measure of precision. This study was primarily designed to test agreement at a stride level, and the average number of strides per trial was suboptimal to test agreement at the trial level. It would be expected that the trial‐level MAE decreases with an increase in strides per trial.

Some limitations apply to this study. Our data reflect 2D measurements of gait asymmetry derived from an estimated groundline which has not been accuracy stress‐tested under field conditions. The algorithm should be compared against an established 3D motion capture or IMU‐based method in future work. However, comparative studies of computer vision deep neural networks with systems that attach devices to the horse can be challenging, as reflective markers or other devices attached to horses could introduce visual artefacts that may fundamentally alter the input to a deep neural network, regardless of whether it was trained with or without markers. This could pose a difficulty in future validation studies.^34^

Overall, the ability to record horses with a basic handheld smartphone and obtain robust gait symmetry measures offers practical advantages: simplified objective lameness detection, longitudinal monitoring and the possibility of telemedicine. This user‐friendly approach aligns with the demand for affordable, field‐ready technologies that reduce reliance on laboratory‐grade setups. With further benchmarking against other gait analysis methods, markerless vision‐based systems may gain widespread acceptance and play a valuable role in everyday lameness examinations and in supporting horse owners' management of equine soundness and welfare.

## 5 CONCLUSION

This study evaluated the agreement between manually annotated and algorithm‐detected keypoints of a vision‐based algorithm developed for equine gait analysis, using data obtained under real‐world field conditions with horses trotting in a straight line or being lunged and recorded using a handheld iPhone. The keypoint agreement between the algorithm and manual annotation demonstrated low bias and narrow error margins. Combined with the minimal variability observed in the annotation versus reannotation substudy, this supports the validity of using manual annotations as a reference standard for evaluating the accuracy of 2D vision‐based algorithms.

The algorithm demonstrated consistently low mean absolute errors and strong agreement between manual annotations and algorithm detections in both stride‐based and trial‐based analyses, showing its ability to detect vertical displacement signals (VDS) and related gait symmetry measures, such as Maxdiff and Mindiff, at the eye, withers and croup. The algorithm estimated groundline showed minimal angular differences compared with the groundline estimated using manually annotated hoof keypoints. Further testing against established objective gait analysis systems is warranted; however, these findings support the algorithm as a reliable field‐based tool for equine gait analysis, demonstrating strong agreement and low variability in keypoint detection and symmetry metrics compared with manual annotation.

## FUNDING INFORMATION

This research was funded by Keydiagnostics ApS as part of the internal validation of their algorithm. No external grants or funding were received.

## CONFLICT OF INTEREST STATEMENT

Authors Karsten Key, Katja Berg and Jakob Kirkegaard are affiliated with Keydiagnostics ApS, a company that provides a commercially available smartphone application “RealHorse” for detecting asymmetry in horses. The computer vision algorithm developed and tested in this study is part of this product. These affiliations may represent a potential conflict of interest, which is hereby disclosed.

## AUTHOR CONTRIBUTIONS

**Karsten Key:** Conceptualization; methodology; writing – original draft; formal analysis; data curation. **Katja Berg:** Methodology; data curation; writing – review and editing; conceptualization. **Jakob Kirkegaard:** Writing – review and editing; software; data curation; methodology; conceptualization. **Kristian Ringkjær Andresen:** Writing – review and editing; data curation. **Sabrina Skov Hansen:** Writing – review and editing.

## DATA INTEGRITY STATEMENT

Karsten Key had full access to all the data in the study and takes responsibility for the integrity of the data and the accuracy of the data analysis.

## ETHICAL ANIMAL RESEARCH

Research ethics committee oversight not currently required by this journal: procedures were performed as part of clinical investigations.

## INFORMED CONSENT

Written owner consent was obtained prior to inclusion in the study.

## Supporting information


**Figure S1.** Eye Maxdiff stride error data for 514 strides comparing algorithm‐detected and manually annotated values. The scatter plot (top‐left) shows close alignment along the line of equality, while the Bland–Altman plot (top‐right) indicates 95% limits of agreement (−8.15 to +8.19 mm). The histogram of signed differences (bottom‐left) reveals minimal bias near zero, and the histogram of absolute differences (bottom‐right) reveals a mean absolute error of 2.93 mm, reflecting generally strong agreement between detected and annotated Eye Maxdiff measurements.

**Figure S2.** Eye Mindiff stride error data for 514 strides comparing algorithm‐detected and manually annotated values. The scatter plot (top‐left) shows close alignment along the line of equality, while the Bland–Altman plot (top‐right) indicates 95% limits of agreement (−7.61 to +7.80 mm). The histogram of signed differences (bottom‐left) reveals a minimal bias near zero (0.09 mm), and the histogram of absolute differences (bottom‐right) shows a mean absolute error of 3.03 mm, reflecting a generally strong agreement between detected and annotated Eye Mindiff measurements.

**Figure S3.** Withers Maxdiff stride error data for 518 strides comparing algorithm‐detected and manually annotated values. The scatter plot (top‐left) shows good alignment along the line of equality, while the Bland–Altman plot (top‐right) indicates 95% limits of agreement at −13.40 to +14.57 mm. The histogram of signed differences (bottom‐left) reveals a near‐zero bias (0.58 mm), and the histogram of absolute differences (bottom‐right) shows a mean absolute error of 5.52 mm, reflecting a generally strong agreement between detected and annotated Withers Maxdiff measurements.

**Figure S4.** Withers Mindiff stride error data for 518 strides comparing algorithm‐detected and manually annotated values. The scatter plot (top‐left) shows good alignment along the line of equality, while the Bland–Altman plot (top‐right) indicates 95% limits of agreement at −13.34 to +14.21 mm. The histogram of signed differences (bottom‐left) reveals a near‐zero bias (0.43 mm), and the histogram of absolute differences (bottom‐right) shows a mean absolute error of 5.46 mm, reflecting a generally strong agreement between detected and annotated Withers Mindiff measurements.

**Figure S5.** Croup Maxdiff stride error data for 524 strides comparing algorithm‐detected and manually annotated values. The scatter plot (top‐left) shows good alignment around the line of equality, while the Bland–Altman plot (top‐right) indicates 95% limits of agreement from −11.71 to +11.16 mm. The histogram of signed differences (bottom‐left) reveals minimal bias near zero, and the histogram of absolute differences (bottom‐right) shows a mean absolute error of 4.33 mm, reflecting generally strong agreement between detected and annotated Croup Maxdiff measurements.

**Figure S6.** Croup Mindiff stride error data for 524 strides comparing algorithm‐detected and manually annotated values. The scatter plot (top‐left) shows good alignment along the line of equality, while the Bland–Altman plot (top‐right) indicates 95% limits of agreement at ±11.80 mm. The histogram of signed differences (bottom‐left) reveals minimal bias near zero, and the histogram of absolute differences (bottom‐right) shows a mean absolute error of 4.38 mm, reflecting generally strong agreement between detected and annotated Croup Mindiff measurements.

## Data Availability

The data that support the findings of this study are openly available in https://doi.org/10.5281/zenodo.15350867.
