# Supplementary material for: Reliability, agreement and variability of a markerless computer vision algorithm for equine gait analysis under field conditions
Source: Equine Vet J. 2025 Nov 4;58(5):1467–81. doi: 10.1111/evj.70109 (PMC13447868; doi:10.1111/evj.70109)
Supplement: Supplementary file 4 [file EVJ-58-1467-s004.pdf]

Equine Veterinary Journal BEVA

Figure S2

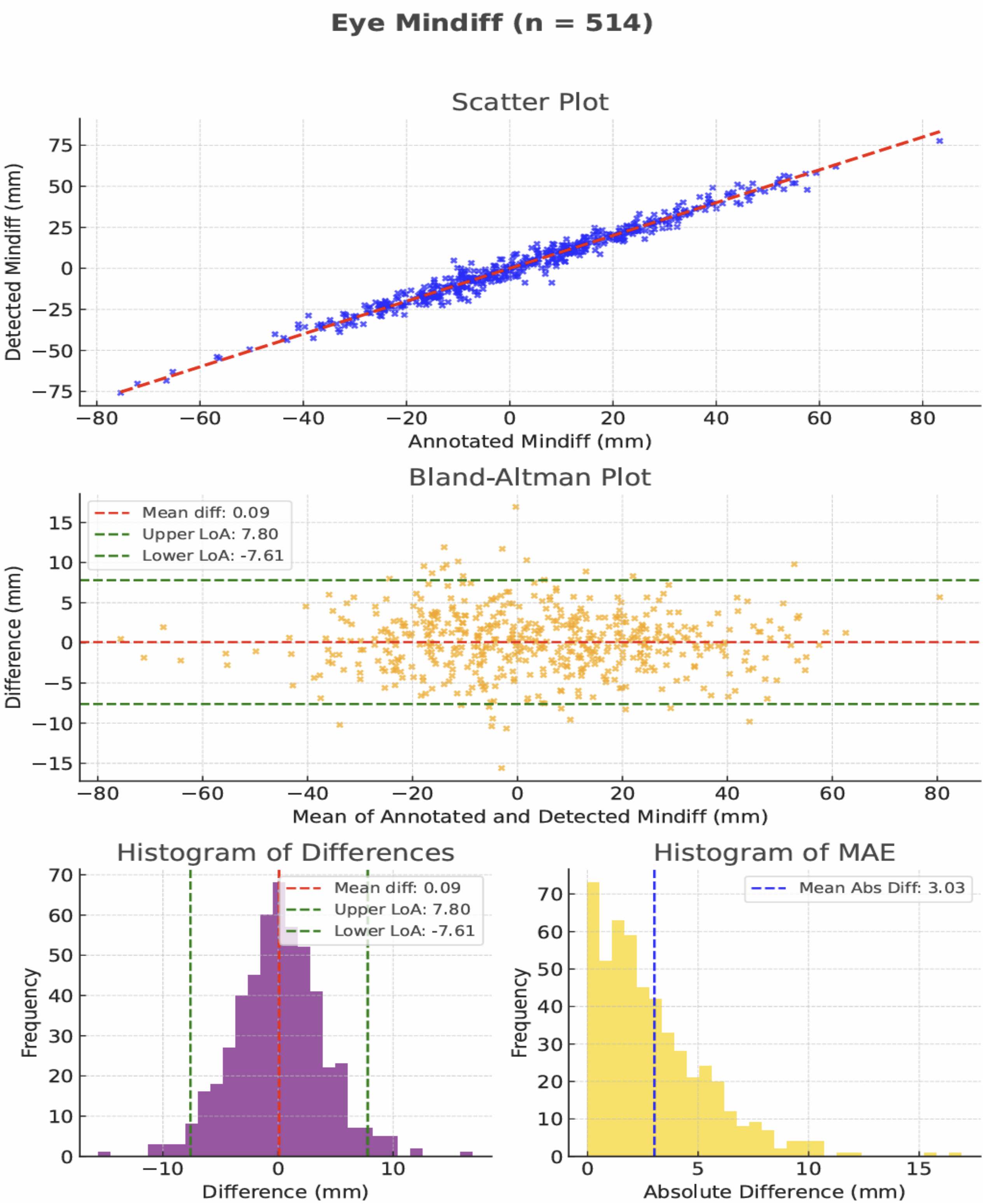

Eye Mindiff stride error data for 514 strides comparing algorithm-detected and manually annotated values. The scatter plot (top-left) shows close alignment along the line of equality, while the Bland-Altman plot (top-right) indicates 95% limits of agreement (-7.61 mm to +7.80 mm). The histogram of signed differences (bottom-left) reveals a minimal bias near zero (0.09 mm), and the histogram of absolute differences (bottom-right) shows a mean absolute error of 3.03 mm, reflecting a generally strong agreement between detected and annotated Eye Mindiff measurements.
