# Supplementary material for: Reliability, agreement and variability of a markerless computer vision algorithm for equine gait analysis under field conditions
Source: Equine Vet J. 2025 Nov 4;58(5):1467–81. doi: 10.1111/evj.70109 (PMC13447868; doi:10.1111/evj.70109)
Supplement: Supplementary file 5 [file EVJ-58-1467-s005.pdf]

Equine Veterinary Journal BEVA

Figure S1

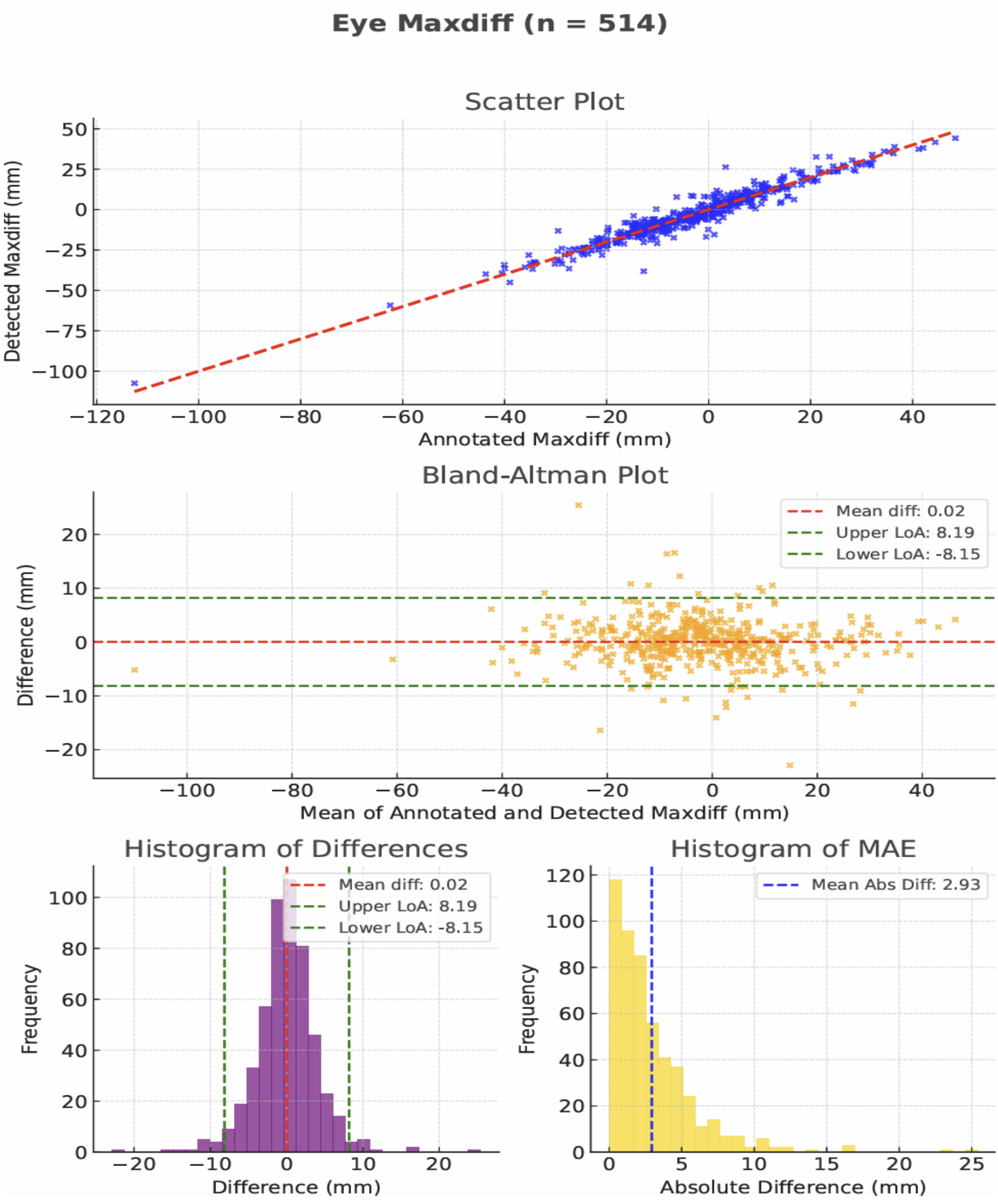

Eye Maxdiff stride error data for 514 strides comparing algorithm-detected and manually annotated values. The scatter plot (top-left) shows close alignment along the line of equality, while the Bland-Altman plot (top-right) indicates 95% limits of agreement (-8.15 mm to +8.19 mm). The histogram of signed differences (bottom-left) reveals minimal bias near zero, and the histogram of absolute differences (bottom-right) reveals a mean absolute error of 2.93 mm, reflecting generally strong agreement between detected and annotated Eye Maxdiff measurements.
